# Supplementary material for: Long-Term Impact of COVID-19 on Hospital Visits of Rural Residents in Guangdong, China: A Controlled Interrupted Time Series Study
Source: Int J Environ Res Public Health. 2022 Oct 14;19(20):13259. doi: 10.3390/ijerph192013259 (PMC9603214; doi:10.3390/ijerph192013259)
Supplement: Supplementary file 1 [file ijerph-19-13259-s001.zip › ijerph-1946866-supplementary.pdf]

Supplementary File

Supplementary File S1: Model specification and coefficient interpretation

Controlled Interrupted Time Series Models

We performed three CITS analyses relatively according to residents' hospitalization visits in different areas, i.e., within-county, out-of-county but within-city, and out-of-city area from April 2017 to March 2021. We also conducted subgroup CITS analyses by gender, age, and levels of the hospital, as well as disease classification of inpatients.

The period from April 2019 to January 2020 was defined as pre-COVID period. The period from February 2020 to March 2021 was defined as COVID-impacted period. The period from April 2017 to January 2018 was defined as equivalent pre-COVID period. The period from February 2018 to March 2019 was defined as equivalent COVID-impacted period. Pre-COVID period and COVID-impacted period was collectively denoted by the implementation period. Equivalent pre-COVID period and equivalent COVID-impacted period was collectively denoted by the control period.

In each stratum, data were fitted using the following equation:

$$Y_t = \beta_0 + \beta_1 \text{ Time} + \beta_2 \text{ Phase} + \beta_3 \text{ Post} + \beta_4 \text{ Group} + \beta_5 \text{ Group}\times\text{Time} + \beta_6 \text{ Group}\times\text{Phase} + \beta_7 \text{ Group}\times\text{Post} + \text{Month} + e_t \quad (1)$$

where "Y_t" is the monthly number of hospital visits. "Time" is the time point of data within a phase (0, 1, 2, 3, ...23). "Phase" indicates "before/after lockdown or equivalent period" (0 refers to the pre-COVID period and the equivalent pre-COVID period, and 1 refers to the COVID-impacted period and the equivalent COVID-impacted period). "Post" indicates the time point after the lockdown, the value is 0 before the lockdown and when the lockdown occurs, and then the value is taken from 1, 2, and so on. "Group" indicates the implementation period (0 for the control period, or 1 for the implementation period). "e_t" denotes the random error.

For Equation 1 (Table S1), interpretation of "β_0" to "β_3" coefficients relate to the comparison group, whereas "β_4" to "β_7" relate to the intervention group. "β_0" is the intercept or number of hospital visits at "Time" = 0. Detailed interpretation of the model coefficients can be seen in Table S1. Table S2 illustrates the data structure for the analyses.

Table S1. Explanation of variables and coefficients in the controlled interrupted time series model

Variable or Coefficient	Explanation
Time	the time point of data within a phase (0, 1, 2, 3, ...23)
Phase	indicates "before/after lockdown or equivalent period" (0 refers to the pre-COVID period and the equivalent pre-COVID period, and 1 refers to the COVID-impacted period and the equivalent COVID-impacted period)
Post	indicates the time point after the lockdown, the value is 0 before the lockdown and when the lockdown occurs, and then the value is taken from 1, 2, and so on
Group	indicates the implementation period (0 for the control period, or 1 for the implementation period)
Group×Time	the time for the implementation period and 0 for the control period. An interaction term to account for the slope difference between the pre-COVID period and equivalent pre-COVID period
Group×Phase	1 for the COVID-impacted period, or 0 for the equivalent pre-COVID, equivalent COVID-impacted and pre-COVID period. An interaction term to account for the difference in the change of levels between the implementation period and control period
Group×Post	the time after lockdown, or 0 for the equivalent pre-COVID, equivalent COVID-impacted and pre-COVID period. An interaction term to account for the difference in the change of slopes between the implementation period and control period
β_1	the pre-COVID slope before February 2018
β_2	the immediate change in monthly hospital visits in 2018, from before February to after February
β_3	trend change (rate of change of monthly hospital visits) in 2018 after February
β_4	the difference in the level between the pre-COVID period and equivalent pre-COVID period
β_5	the slope difference between the pre-COVID period and equivalent pre-COVID period
β_6	instantaneous effect of COVID-19. The difference in the change of levels between the implementation period and control period.
β_7	long-term effect of COVID-19. The difference in the change of slopes between the implementation period and control period
e_t	denotes the random error

Implementation period: period from April 2019 to March 2021; control period: period from April 2017 to March 2019; pre-COVID period: period from April 2019 to January 2020; COVID-impacted period: period form February 2020 to March 2021; equivalent pre-COVID period: period from April 2017 to January 2018; equivalent COVID-impacted period: period from February 2018 to March 2019

Table S2. Structure of data for analysis of the impact of COVID-19 lockdown on hospital visits within the county. The dependent variable is monthly hospital visits

Implementation period								Control period							
Y_t	Time (month)	Phase	Post	Group	Group× Time	Group× Phase	Group× Post	Y_t	Time (month)	Phase	Post	Group	Group× Time	Group× Phase	Group× Post
2567	0	0	0	1	0	0	0	3562	0	0	0	0	0	0	0
2925	1	0	0	1	1	0	0	3799	1	0	0	0	0	0	0
2682	2	0	0	1	2	0	0	3674	2	0	0	0	0	0	0
2902	3	0	0	1	3	0	0	3862	3	0	0	0	0	0	0
2847	4	0	0	1	4	0	0	3612	4	0	0	0	0	0	0
2367	5	0	0	1	5	0	0	3357	5	0	0	0	0	0	0
2626	6	0	0	1	6	0	0	3504	6	0	0	0	0	0	0
2632	7	0	0	1	7	0	0	3293	7	0	0	0	0	0	0
3023	8	0	0	1	8	0	0	3611	8	0	0	0	0	0	0
2203	9	0	0	1	9	0	0	3714	9	0	0	0	0	0	0
2584	10	1	0	1	10	1	0	2204	10	1	0	0	0	0	0
2925	11	1	1	1	11	1	1	2350	11	1	1	0	0	0	0
2902	12	1	2	1	12	1	2	2579	12	1	2	0	0	0	0
3255	13	1	3	1	13	1	3	2470	13	1	3	0	0	0	0
3087	14	1	4	1	14	1	4	2666	14	1	4	0	0	0	0
3122	15	1	5	1	15	1	5	2837	15	1	5	0	0	0	0
3138	16	1	6	1	16	1	6	3053	16	1	6	0	0	0	0
3070	17	1	7	1	17	1	7	3156	17	1	7	0	0	0	0
3344	18	1	8	1	18	1	8	2837	18	1	8	0	0	0	0
3333	19	1	9	1	19	1	9	2816	19	1	9	0	0	0	0
3950	20	1	10	1	20	1	10	2886	20	1	10	0	0	0	0
2990	21	1	11	1	21	1	11	3043	21	1	11	0	0	0	0
2858	22	1	12	1	22	1	12	1981	22	1	12	0	0	0	0
3632	23	1	13	1	23	1	13	2314	23	1	13	0	0	0	0

Implementation period: period from April 2019 to March 2021; control period: period from April 2017 to March 2019
